# Gastric emptying scintigraphy in postprandial distress syndrome

**DOI:** 10.12669/pjms.341.14137

**Published:** 2018

**Authors:** Muhammad Hafeez, Fida Hussain, Amjad Salamat, Muhammad Bilal Khan

**Affiliations:** 1 Dr. Muhammad Hafeez, Consultant Physician & Gastroenterologist, Combined Military Hospital Multan Pakistan; 2 Dr. Fida Hussain, Department of Nuclear Medicine, Armed Forces Institute of Pathology Rawalpindi Pakistan; 3 Dr. Amjad Salamat, Professor of Gastroenterology and Consultant Physician, Quaid-e-Azam International Hospital, Rawalpindi, Pakistan; 4 Mr. Muhammad Bilal Khan, Statistician, NMC, AFIP, Rawalpindi, Pakistan

**Keywords:** Gastric emptying scintigraphy, Post prandial distress syndrome, Functional Dyspepsia

## Abstract

**Objective:**

To find out the pattern of gastric emptying scintigraphy (GES) in patients with post prandial distress syndrome (PDS).

**Methods:**

This study was carried out from January 2015 to July 2016 at Combined Military Hospital (CMH) Kharian and Nuclear Medical Centre (NMC) of Armed Forces Institute of Pathology (AFIP) Rawalpindi. Patient's inclusion criteria were dyspepsia of post prandial distress type for more than six months duration. Patients with dyspepsia due to epigastric pain syndrome and other organic disorder were excluded. Upper gastrointestinal endoscopy was performed in all patients to rules out organic causes. Four-hour Gastric emptying scintigraphy was carried out at NMC, AFIP. Results were compiled and statistical assessment was done by utilizing SPSS IBM 22 version.

**Results:**

Thirty-eight patients were included in the study with age range from 15-72 years with mean age of 37.05±13.5 years. Males were 28(73.7%) and 10(26.7%) were female. Mean gastric retention with SD at one, two, three and four hours were 63 ± 19.04, 37± 20.62, 19±16.66 and 10±12.73 percent respectively. Early gastric emptying was in 3(7.89%) and delayed gastric emptying at two and four hours was seen in 4(10.52%) and 12(32%) respectively. Seventeen (44%) of the patients had normal gastric emptying despite the classical symptoms of PDS.

**Conclusion:**

Gastric dysmotility in GES seen in half of the patients points some additional mechanism as well like gastric accommodation or visceral hypersensitivity in the patients with PDS.

## INTRODUCTION

Dyspepsia is derived from two Greek words, “Dys” means bad and “Pepsis” means digestion. Patients may present with different symptoms like bloating, anorexia, early satiety, epigastric discomfort. By definition non-ulcer dyspepsia is epigastric symptom in the absence of structural lesion.^1^ According to Rome III criteria non-ulcer dyspepsia is one of the three symptoms for last three months with onset at least six months previously. The symptoms are postprandial fullness, early satiety, and epigastric pain or burning.^2^ Different mechanisms of non-ulcer dyspepsia have been postulated that includes visceral hypersensitivity, delayed gastric emptying and psychological stress.^3^ In irritable bowel syndrome, upper and lower gut dysmotility are the same.^4^

There are multiple ways to establish the diagnosis by quantifying the gastric emptying. One of the well-established non-invasive and quantitative method for determination of gastric emptying is Gastric emptying scintigraphy (GES).^5^ By this method retention of radiolabeled food in stomach is measured with reference to post intake time. Abnormal gastric emptying has been more linked to post prandial fullness, nausea and vomiting.^6^ Early and delayed gastric emptying produces the similar symptoms, so it's important to find out both abnormal patterns in the single study. Several professional societies like Society of Nuclear Medicine [SNM], American Gastroenterological Association (AGA), and the American Neuro gastroenterology and Motility Society (ANMS) have developed guidelines for performing GES. They have jointly formulated the guidelines of performing and interpretation of the GES studies. Four hours GES is recommended now a days because of variable patterns being observed at two and four hours on radionuclide gastric emptying studies.^7^ Delayed gastric emptying is one of the most reported pathophysiological mechanisms in post prandial distress patients.^8^,^9^ However, these guidelines could not be implemented internationally because of multiple geographical and non-geographical factors. Multi-centric international studies are required to establish an international guideline. This study was designed to see the percentage retention of food in the stomach at one, two, three and four hours in patients with post prandial distress syndrome.

## METHODS

This study was carried out at Combined Military Hospital (CMH) Kharian from January 2015 to June 2016 after approval by the ethical committee of the hospital. A total 38 patients were selected by consecutive convenient sampling. Patients were inquired in detail about warning symptoms; like reduced appetite, weight loss, and GI bleed. Patients were selected on the basis of Rome III criteria with symptoms, like post prandial fullness, bloating, distension, nausea, vomiting and early satiation. Informed consent was taken from each patient. Patients with history suggestive of epigastric pain syndrome, diabetes mellitus, not willing to leave smoking, drugs causing dysmotility like Domparidone, Itopride, pain medications and who had undergone gastric surgery were excluded from the study.

In females GES was performed during 1^st^ week of menstrual cycle to avoid influence from hormonal misbalance. In all these selected patients, upper GI Endoscopy was done after overnight fast to rule out any structural lesion like ulcer, growth or any other anomaly. Gastric emptying scintigraphy was performed at Nuclear Medical Centre Armed Forces Institute of Pathology (NMC, AFIP). The study was performed by standard GES protocols. Technetium Tc-99m sulfur colloid (0.5–1 mCi) labeled meal consisting of two large eggs white, two slices of bread and 30 grams jam with 120 ml of water was given to the patient. Imaging was performed using large field of view Siemens ECAM^®^ dual head gamma camera. Imaging properties were set to 64×64 matrix by utilizing low energy all-purpose collimator. The photo peak settings were 20% around the 140-keV peak for ^99m^Tc. Anterior and posterior planar images of upper abdomen by including distal esophagus, stomach, and proximal small bowel in the field of view acquired for one-minute, immediately after ingestion of the radiolabeled meal. Repeated static images of one-minute duration were acquired in similar anatomy at hourly intervals till four hours post intake. Region of interest (ROI) was drawn around the activity in the entire stomach in anterior and posterior views. All data was corrected for radioactive decay.

The geometric mean activity of decay corrected counts (square root of the product of the anterior and posterior counts) was determined for each time-based image. The final measurement of gastric emptying was based on the percentage of gastric retention at specific times after meal ingestion. The normal internationally accepted values for low-fat, egg-white gastric emptying scintigraphy are summarized in Table-I.^10^

**Table-I:** Normal value for Low-Fat, Egg-White Gastric retention.

Time point (in hours)	Lower limit (a lowervalue suggestsabnormally rapidgastric emptying)	Upper limit (agreater value suggests abnormally delayedgastric emptying)
0.5h	70%	
1.0h	30%	90%
2.0h		60%
3.0h		30%
4.0h		10%

Grading for severity of delayed gastric emptying based on four hour value in groups related to the standard deviation of the normal results^11^:

1. Grade 1 (mild): 11-20% retention at 4 hours
2. Grade 2 (moderate): 21-35% retention at 4 hours
3. Grade 3 (severe): 36-50% retention at 4 hours
4. Grade 4 (very severe):>50% retention at 4 hours

### Statistical Analysis

Statistical analysis was performed using the SPSS-22. Descriptive statistics were used to describe this information and data. Chi square test was applied to compare qualitative variables between the symptoms and gastric retention at different intervals. Independent samples t-test was used to compare duration of symptoms and percentage of retention in “symptoms duration” based groups. Continuous and categorical variables are reported as mean ± SD and percentages, respectively. A p-value <0.05 was considered as significant.

## RESULTS

Thirty eight patients were included in our study with age ranges from 15 to 72 years with mean age of 37.05±13.5 years. Among these 38 patients 28 (73.3%) were male and 10 (26.7) were female. The duration of symptoms in these 38 patients are summarized in Table-II. However, for comparison with gastric retention we divided these patients in three groups on the basis of their symptoms Table-II. Group-I consisting of 12 patients who had symptoms of one-year duration, Group-II 12 patients have 1-3 years and Group-III 14 patients have symptoms more than three years duration.

**Table-II:** Duration of post prandial distress syndrome with frequency.

Groups	Duration of symptoms	Frequency	Group Frequency	Group percentage
Group-I	6 months	7(18.4%)	12	31.58
	1 year	5(13.2%)		
Group-II	1 and half year	4(10.5%)	12	31.58
	2 years	7(18.4%)		
	2 and half year	1(2.6%)		
Group-III	More than 3 years	14(36.8%)		36.84
	Total	38		100

### Gastric Emptying Scintigraphy Results

In our study stomach counts at hourly intervals represent the percentage amount of food retention in the stomach. At one-hour post intake the gastric retention ranges from 15% to 98% with mean retention of 62.3%±18.6. At two hours the values range from 2% to 75% with mean value of 36.94%±20.49. There was 1% to 60% and 0% to 48% food retention in stomach at three and four hours post intake respectively with mean retention of 18.73%±16.7 and 10.40%±12.74 respectively. The data is summarized in Table-III. In our study population the results revealed that from one to three hours there was significantly low food retention in the stomach as compared to the internationally accepted 90%, 60% and 30% values. However, at 4 hours our results are comparable with the international standards.^7^

**Table-III:** Mean gastric food retention hourly in total population.

Gastric Retention at	Mean	Standard Deviation	*p*-value
1 hour	63.98	19.04	<0.01*
2 hours	36.87	20.62	<0.01*
3 hours	18.77	16.66	<0.01*
4 hours	10.42	12.73	0.839

* p<0.05 considered significant using one sample t-test.

### Number of cases vs Early or delayed Gastric Emptying

With reference to normal gastric retention as given in Table-I, there were three cases of early gastric emptying among all these 38 patients who had less than 30% food retention at one-hour post ingestion. The delayed gastric emptying with stomach food retention of more than 90% at one hour two cases and at two hours more than 60% retention seen in four patients. However, at four hours post ingestion delayed gastric emptying with more than 10% stomach food retention noted 12 patients and among these 12 patients five had mild, four had moderate and three have sever delayed gastric retention based on the severity grading given in material and methods section. The percentage values are summarized in Table-IV.

**Table-IV:** Early or delayed Gastric Emptying and number of cases.

Time after intake	Early gastric emptying	Percentage	Delayed gastric emptying	Percentage
1 hour	3	7.89	2	5.26
2 hours	-		4	10.52
4 hours	-		12	31.57
			Mild = 4	13.15
			Moderate = 4	10.52
			Severe = 3	7.89

### Gastric retention with respect to duration of symptoms

The patients were divided in three groups on the basis of duration of symptoms Table-II. The duration of symptoms correlated with gastric retention Table-V. There is no significant difference in the mean gastric retention between Group-I and Group-II at one, two, three and four hours. However, there is statistically significant difference in mean gastric retention between Group-III and rest of the two Groups at 2, 3 and 4 hours post intake Table-VI.

**Table-V:** Hourly Mean±SD gastric food retention in each group on the basis of symptoms.

Groups	Gastric retention at 1 hr	Gastric retention at 2 hr	Gastric retention at 3 hr	Gastric retention at 4 hr
Group-I one year	61.96±13.49	31.23±21.63	15.77±20.85	8.96±17.38
Group-II 1 to 3 years	60.42±20.57	30.29±19.385	14.78±14.229	7.71±9.093
Group-III ≥ 3 years	69.31±19.42	47.69±18.221	25.11±16.472	14.45±13.860
Total	63.98±19.04	36.87±20.623	18.77±16.662	10.42±12.733

**Table-VI:** Hourly comparisons of means among groups.

Dependent Variable	(I) Duration of symptom of the pt	(J) Duration of symptom of the pt	Mean Difference (I-J)	Std. Error	p-value
Gastric retention at 1 hr	One year or less	1 to 3 Years	2.808	7.788	0.721
		More than 3 years	-7.039	7.505	0.355
	1 to 3 Years	More than 3 years	-9.848	7.505	0.198
Gastric retention at 2 hr	One year or less	1 to 3 Years	10.972	7.690	0.163
		More than 3 years	-11.642	7.411	0.125
	1 to 3 Years	More than 3 years	-22.614*	7.411	0.004
Gastric retention at 3 hr	One year or less	1 to 3 Years	11.672	6.386	0.076
		More than 3 years	-4.204	6.154	0.499
	1 to 3 Years	More than 3 years	-15.876*	6.154	0.014
Gastric retention at 4 hr	One year or less	1 to 3 Years	7.684	5.017	0.135
		More than 3 years	-2.530	4.835	0.604
	1 to 3 Years	More than 3 years	-10.214*	4.835	0.042

## DISCUSSION

Symptomatically patients with early gastric emptying presents in the similar way as delayed gastric emptying. We observed one hour early gastric emptying in 3(7.89%) and delayed gastric emptying in 2(5.26%) patients. This is significantly low as reported by Delgado et al. in which early gastric emptying was seen in 41% of the patients.^12^ According to consensus guidelines upper limit of food retention at two and four hours is>60 and >10% respectively, that is considered to be delayed gastric emptying. In our study 10.52 % patients had delayed gastric emptying at two hours. At four hours mild, moderate and severe food retention was present in 13.15%, 10.52% and 7.89% respectively with 12 (32%) patients in total. This observation is close to Japanese study by Asano H et al. where gastric emptying was 25.5% in PDS group of FD.^13^

Overall our 44.7% patients showed normal gastric emptying despite classical symptoms pointing some different mechanism for PDS like visceral hypersensitivity or gastric accommodation other than delayed gastric emptying. Ochi M et al also did not find any association of delayed gastric emptying in the two groups of functional dyspepsia pointing to different mechanism for PDS.^14^ This is supported by a recent study by Vanheel et al.^15^ where visceral hypersensitivity was closely associated with PDS. Further they did not find any difference between the Rome-III subgroups in the prevalence of gastric hypersensitivity, impaired gastric accommodation and delayed gastric emptying. However delayed gastric emptying was more observed in the overlap group. Concept of hypersensitivity with PDS has also been supported in an early study by Tack J et al.^16^ and recent study by Di Stefano et al.^17^ Moreover, we presented correlation of duration of symptoms with the gastric retention. The data showed that there is linear correlation of duration symptoms with the amount of food retention in the stomach. As evident by delayed gastric emptying in patients who had symptoms for three or more duration. Our study supports the emerging concept of mixed pathophysiology rather than previous concept of delayed gastric emptying in PDS. This will help in treating the patients with drugs focusing to all angles of pathophysiology rather treating with prokinetics only, as only few of them may lead to serious cardiac arrhythmias. Our study showed similar and matching results because of the standardized procedure and guidelines form PDS by SNM, AGA and ANMS societies were applied. Epidemiological, racial or life style may have played a role as associated factors in different presentations of our study. Needs more studies on these lines.

### Limitations of the study

Due to long waiting list of GES and prolong procedure time, few patients agreed to participate in this study.

## CONCLUSION

Visceral hypersensitivity, gastric accommodation may be pathophysiological mechanism other than delayed gastric emptying in PDS. More studies are suggested to strengthen this concept.

### Authors' Contributions

**MH** conceived, designed and did statistical analysis & editing of manuscript.

**AS** designed & refined the idea, helped in data collection and final editing of the manuscript.

**FH, MB** looked at the nuclear section, designing, editing and statistical analysis.

**MB** also covered the nuclear section, designing, editing and statistical analysis.

## References

[1] Tack J, Talley NJ, Camilleri M (2006). Functional gastro duodenal disorders. Gastroenterology.

[2] Tack J, Talley NJ (2013). Functional dyspepsia - symptoms, definitions and validity of the Rome III criteria. Nat Rev Gastroenterol Hepatol.

[3] Timmons S (2004). Functional dyspepsia: motor abnormalities, sensory dysfunction and therapeutic options. Am J Gastroentrol.

[4] Thompson WG (1984). Non-ulcer dyspepsia. Can Med Assoc J.

[5] Seok JW (2011). How to Interpret Gastric Emptying Scintigraphy. Neurogastroenterol Motil.

[6] Sarnelli G, Caenepeel P, Geypens B (2003). Symptoms associated with impaired gastric emptying of solids and liquids in functional dyspepsia. Am J Gastroenterol.

[7] Abell TL, Camilleri M, Donohoe K, Hasler WL, Lin HC, Maurer AH (2008). Consensus Recommendations for Gastric Emptying Scintigraphy: A Joint Report of the American neurogastroenterology and motility Society and the society of nuclear medicine. Am J Gastroenterol.

[8] Shindo T, Futagami S, Hiratsuka T (2009). Comparison of gastric emptying and plasma ghrelin levels in patients with functional dyspepsia and non-erosive reflux disease. Digestion.

[9] Karamanolis G, Caenepeel P, Arts J, Tack J (2006). Association of the predominant symptom with clinical characteristics and pathophysiological mechanisms in functional dyspepsia. Gastroenterology.

[10] Abell TL, Camilleri M, Donohoe K, Hasler WL, Lin HC, Maurer AH (2008). Consensus recommendations for gastric emptying scintigraphy: a joint report of the American neurogastroenterology and motilitysociety and the society of nuclear medicine. J Nucl Med Technol.

[11] (2007). Consensus Recommendations for Gastric Emptying Scintigraphy A Joint Report of The Society of Nuclear Medicine and The American Neurogastroenterology and Motility Society, April 23. http://interactive.snm.org/docs/GES_Consensus_Manuscript_4-23a-2007.pdf.

[12] Delgado-Aros S, Camilleri M, Cremonini F, Ferber I, Stephens D, Burton DD (2004). Contributions of gastric volumes and gastric emptying to meal size and post meal symptoms in functional dyspepsia. Gastroenterology.

[13] Asano H, Tomita T, Nakamura K, Yamasaki T, Okugawa T, Kondo T (2017). Prevalence of gastric motility disorders in patients with functional dyspepsia. J Neurogastroenterol Motil.

[14] Ochi M, Tominaga K, Tanaka F (2013). Clinical classification of subgroups according to the Rome III criteria cannot be used to distinguish the associated respective pathophysiology in Japanese patients with functionaldyspepsia. Intern Med.

[15] Vanheel H, Carbone F, Valvekens L, Simren M, Tornblom H, Vanuytsel T (2017). Pathophysiological Abnormalities in Functional Dyspepsia Subgroups According to the Rome III Criteria. Am J Gastroenterol.

[16] Tack J, Caenepeel P, Fischler B (2001). Symptoms associated with hypersensitivity to gastric distention in functional dyspepsia. Gastroenterology.

[17] Di Stefano M, Miceli E, Tana P (2014). Fasting and postprandial gastric sensorimotor activity in functional dyspepsia: postprandial distress vs epigastric pain syndrome. Am J Gastroenterol.

